# Renaissance of Phoenix Drug Thalidomide—New Insights into Practical Clinical Application and Optimization Strategies for Managing Adverse Effects in Digestive Diseases

**DOI:** 10.3390/ph18111689

**Published:** 2025-11-07

**Authors:** Xiaxiao Yan, Ziqi Guo, Chengzhen Lyu, Rou Tang, Rutong Li, Hongwei Wang, Kai Song, Wangyang Chen, Kun He, Dong Wu

**Affiliations:** 1 Department of Emergency, State Key Laboratory of Complex Severe and Rare Diseases, Peking Union Medical College Hospital, Chinese Academy of Medical Sciences and Peking Union Medical College, Beijing 100730, China; 2 Department of Gastroenterology, State Key Laboratory of Complex Severe and Rare Diseases, Peking Union Medical College Hospital, Chinese Academy of Medical Sciences and Peking Union Medical College, Beijing 100730, China; 3 Department of Pharmacy, Peking Union Medical College Hospital, Chinese Academy of Medical Sciences & Peking Union Medical College, Beijing 100730, China; 4 Department of Gastroenterology, People’s Hospital of Xizang Autonomous Region, Lhasa 850000, China

**Keywords:** thalidomide, digestive diseases, application, adverse effects, drug delivery

## Abstract

Thalidomide, once withdrawn due to teratogenicity, has re-emerged as a repurposed agent to treat a broad spectrum of diseases because of its anti-inflammatory, immunomodulatory, and anti-angiogenic properties. Over the past two decades, thalidomide has also been increasingly used in digestive diseases, including gastrointestinal and hepatobiliary–pancreatic disorders. Despite these expanding indications, its broader clinical use remains restricted by safety concerns and the absence of standardized, evidence-based guidance. In particular, practical strategies for optimizing efficacy while minimizing toxicity have not been systematically summarized. This review aims to provide an updated, integrated, and clinically oriented overview of thalidomide in digestive diseases, summarizing the expanded applications from immune-mediated to non-immune-related conditions, highlighting real-world applications, therapeutic strategies, and approaches to risk mitigation. Furthermore, we discuss structural analogs and novel delivery systems that may enhance safety and efficacy, paving the way for thalidomide’s rational and safe use as a modern “phoenix drug”.

## 1. Introduction

Thalidomide was synthesized in 1954 and prescribed as a sedative, tranquilizer, and antiemetic for treating morning sickness in pregnant women [1]. Thalidomide was marketed in over 40 countries worldwide but was withdrawn from the market because of a wide range of serious teratogenic effects on fetuses, including characteristic limb defects [2,3]. The median lethal dose (LD_50_) of thalidomide varies among species and administration routes: 113 mg/kg orally in rats, 1550 mg/kg dermally in rats, and approximately 2000 mg/kg orally in mice, indicating an overall low acute toxicity despite its severe teratogenicity [4].

**Figure 1:**
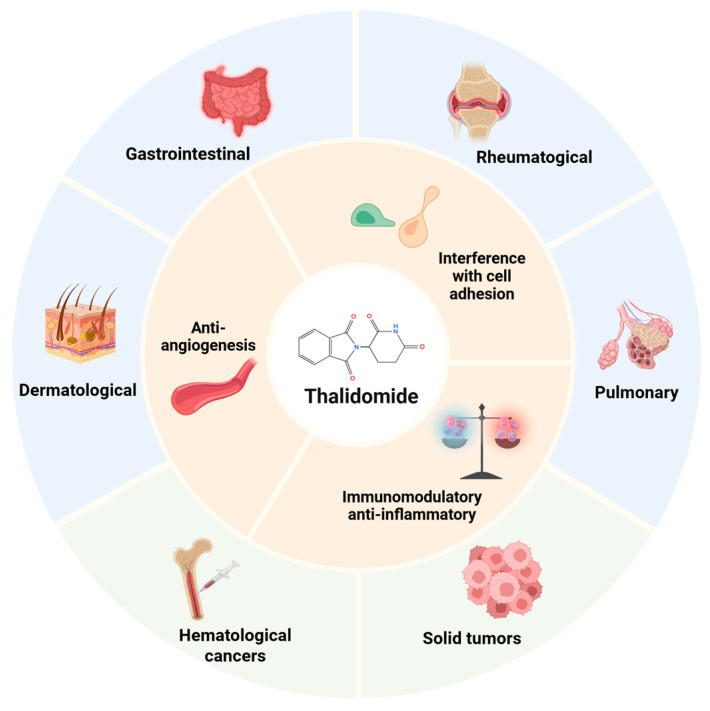
Therapeutic applications of thalidomide. Multifaceted mechanism of thalidomide and its clinical applications in malignant diseases and inflammatory, autoimmune, and infectious conditions. Created in BioRender. Chen, W. (2025) https://BioRender.com/7t7ppxs.

Surprisingly, subsequent studies have shown that thalidomide has unexpected clinical benefits, allowing this “phoenix drug” to re-enter the market. Since thalidomide exited the market in 1961, this drug has been proven to be effective at inhibiting angiogenesis, immunomodulation, and interfering with cell adhesion, and has been increasingly used in the management of various diseases (Figure 1). Thalidomide’s resurgence came about from the serendipitous discovery in 1965 of its surprising activity against erythema nodosum leprosum lesions, and in 1998, the FDA approved it for this indication [5]. Its antiangiogenic effect was discovered in 1994, which is mediated by inhibiting fibroblast growth factor- and vascular endothelial growth factor (VEGF)-induced angiogenesis, which contribute to fetal deformities but also endow it with antitumor activity [1,6]. Thalidomide has been tested to treat a range of hematologic malignancies, and in 2006, the FDA approved it for a new indication of multiple myeloma [7,8,9]. Numerous studies have also confirmed the therapeutic activity and clinical promise of thalidomide in solid tumors, including prostate cancer, Kaposi’s sarcoma, malignant melanoma, colorectal cancer, and renal cell carcinoma [10,11]. Recently, the anti-inflammatory and immunomodulatory actions of thalidomide have made it a potential therapy for treating autoimmune and autoinflammatory diseases, such as systemic lupus erythematosus, Behcet’s disease, and rheumatoid arthritis [12,13,14,15,16].

In recent years, thalidomide has also been increasingly used in digestive diseases, including both gastrointestinal (GI) and hepatobiliary–pancreatic disorders. Thalidomide has shown promise in immune-related digestive diseases, such as inflammatory bowel disease (IBD), intestinal Behcet’s disease (intestinal BD), and Immunoglobulin G4 (IgG4)-related disease. It is also a valuable agent in non-immune-related conditions, including GI bleeding and digestive malignancies. The expanding indications reflect its broad therapeutic potential in gastroenterology. However, the clinical use of thalidomide remains limited by concerns over adverse effects, particularly peripheral neuropathy and teratogenicity. Mitigation of these adverse effects is a necessary step toward wider and safer clinical use of this promising agent. Therefore, this review aims to provide an updated and integrated summary of the clinical applications of thalidomide in digestive disorders and to propose strategies for safer and more effective use of this drug. An emphasis is placed on real-world clinical strategies (alternative, salvage, and combination therapies), practical dosing guidance and, for the first time, comprehensive optimization approaches including dose management, structural modification, and targeted delivery systems. These discussions collectively aim to facilitate the renaissance of thalidomide as a repurposed “phoenix drug” in modern gastroenterology.

## 2. Mechanisms of Thalidomide

**Figure 2:**
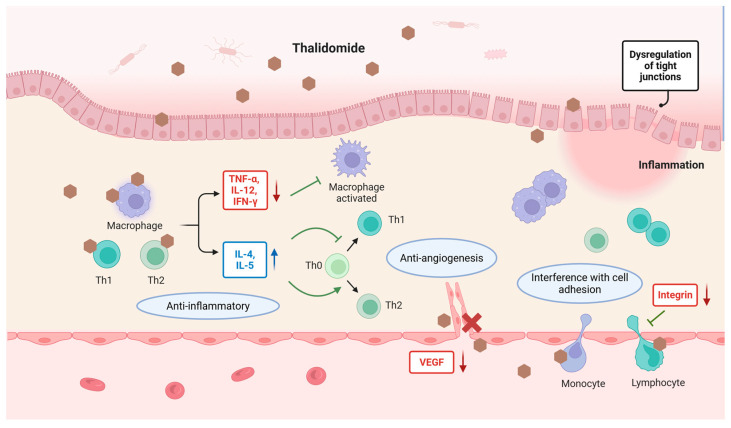
Mechanisms of thalidomide’s therapeutic effects in digestive diseases. Thalidomide inhibits proinflammatory cytokines such as TNF-α, IL-12, and IFN-γ while enhancing the anti-inflammatory cytokines IL-4 and IL-5. It modulates T cell differentiation, reduces macrophage activation, and interferes with cell adhesion by downregulating integrin expression. In addition, thalidomide inhibits VEGF-mediated angiogenesis. These diverse mechanisms contribute to its therapeutic efficacy across a range of digestive conditions. Created in BioRender. Chen, W. (2025) https://BioRender.com/taojw9a.

Thalidomide has multiple underlying anti-inflammatory and immunomodulatory mechanisms, including blocking the alternative activation of macrophages and activation of the NF-κB pathway; downregulating tumor necrosis factor (TNF)-α, interferon (IFN)-γ, and interleukin (IL)-12; promoting IL-4 and IL-5 production; interfering with integrin expression and integrin-mediated signaling pathways; reducing circulating T cells; reducing leukocyte adhesion to endothelial cells; and inhibiting VEGF-related angiogenesis [1,17,18,19,20,21,22,23]. These diverse mechanisms have been implicated in thalidomide’s therapeutic effects in digestive diseases (Figure 2). Given these clinical benefits and widespread applications, it is unsurprising that thalidomide is enjoying a renaissance. Until recently, new uses of this old drug have continued to be supported by research findings, including its use in COVID-19, idiopathic pulmonary fibrosis, small-intestinal angiodysplasia, and radiation-induced blood–brain barrier injury [24,25,26,27]. In 2010, cereblon (CRBN) was identified as the direct protein target of thalidomide, which mediates its teratogenic and anti-myeloma effects [28,29]. In the ubiquitin–proteasome system (UPS), the specificity of substrate or target protein recognition is determined by the type of E3 ubiquitin ligase. Human cells express nearly a thousand distinct E3 ubiquitin ligases, among which CRBN serves as the substrate-recognition subunit of a multi-subunit E3 ligase complex (CRL4). Thalidomide, the first discovered protein degrader, hijacks the UPS protein degradation machinery. By binding to CRBN, it generates a novel substrate-binding interface that selectively recruits and degrades multiple neosubstrates. Thus, after half a century of research, the understanding of thalidomide’s mechanism has evolved from a “multi-target, pleiotropic” model to a “single-target, pleiotropic” paradigm. Depending on the specific neosubstrate involved, thalidomide exhibits diverse biological activities including teratogenicity, immunomodulation, angiogenesis inhibition, and myeloma cell killing.

## 3. Thalidomide in Digestive Diseases: Clinical Applications and Limitations

The potential of thalidomide in digestive diseases was first recognized in 1979 when Waters et al. reported its efficacy in a patient with refractory ulcerative colitis [30]. Since then, thalidomide has been increasingly investigated for its immunomodulatory and anti-angiogenic effects in various digestive conditions. However, beyond teratogenic concerns, its clinical use is also restricted by its other adverse effects. Careful dose management, including the use of lower doses or intermittent regimens, has improved its tolerability and extended its potential use to selected patients. The digestive system disorders in which thalidomide has been used can be broadly categorized into immune-related and non-immune-related conditions. The following sections provide an overview of how thalidomide has been used over the years in a range of digestive disorders (Table 1), focusing on its clinical applications, therapeutic value, and remaining challenges.

### 3.1. Immune-Related Digestive Diseases

As mentioned above, thalidomide exerts multiple anti-inflammatory and immunomodulatory effects, which explains its increasing application in immune-related diseases in recent years. Among digestive system diseases, thalidomide is mainly used in intestinal inflammatory diseases, IgG4-related digestive diseases, and oral mucosal disorders.

#### 3.1.1. Intestinal Inflammatory Diseases

Broadly defined, intestinal inflammatory diseases include conditions such as ulcerative colitis, Crohn’s disease, and intestinal Behcet’s disease. Over the years, thalidomide has been applied in both adult and pediatric populations with these conditions. Among all the digestive diseases it is used in, this group represents the most extensively studied and clinically utilized indication for thalidomide. Despite the emergence of novel therapies [48], thalidomide still retains a valuable role in selected cases. Its roles in modulating immune responses, suppressing inflammation, and promoting mucosal healing have drawn increasing attention, though limitations such as potential side effects still require considerations.

##### Ulcerative Colitis and Crohn’s Disease in Adults

The efficacy of thalidomide in refractory inflammatory bowel disease was first reported in ulcerative colitis in 1979 and later in Crohn’s disease in 1997 [30,49]. Two open-label pilot studies published in 1999 suggested its potential efficacy in refractory CD [50,51]. In 2016, a systematic review including 31 studies and 489 patients comprehensively analyzed various outcomes. Clinical responses and remission were reported in 80.0% patients at 6 months and 72.2% at 12 months and 71.7% patients were able to reduce their steroid dosage. Fistulae improved in 60.5% of cases and closed in 34.6%. Endoscopic improvement was observed in 69.7% of patients and complete mucosal healing was observed in 53.0% patients. The cumulative incidence of total adverse events and those leading to drug suspension were 75.6 and 19.7/1000 patient months, respectively. Neurological disturbances accounted for 64.3% of the adverse events and were the most frequent cause of drug withdrawal [52].

In some rarer or even extreme situations with failure of conventional treatment, physicians thought of thalidomide and obtained favorable therapeutic effects. Esophageal involvement in CD is rare but responds to thalidomide treatment, manifesting as improved esophageal pain or dysphagia and the healing of endoscopic esophagitis [53,54]. CD-associated refractory orofacial granulomatosis, oral aphthous ulcers, and vulvar ulcers all showed remission after thalidomide treatment [55,56,57,58]. Apart from inflammation in the intestinal tract, mesenteric panniculitis also responded well to thalidomide in a prospective open-label pilot study [59,60].

Thalidomide has also shown satisfactory performance during long-term observation but attention should be paid to safety issues as the cumulative drug dosage increases. A retrospective multicenter study observed clinical remission after thalidomide treatment in the first year in 54% of patients with refractory active intestinal or perineal CD. However, 46% of patients withdrew thalidomide at 24 months because of toxicity [61]. In a cohort of 69 CD patients from China, 68 achieved complete clinical remission. The long-term remission rate at 60 months exceeded 50% [62]. In a randomized controlled trial (RCT) in China, the clinical remission rate in the thalidomide group was significantly higher at the 8-week (68%) and 48-week follow-ups (46.3%) [63]. Although the incidence of adverse events was high in several of the above studies during the treatment, most were mild and well tolerated. The most common adverse events included drowsiness, constipation, rash, and peripheral neuropathy. Rare but severe adverse events included thrombosis, acute onset of psychotic symptomatology, severe postural hypotension, sinus bradycardia, and bowel perforation that were reported in some specific cases [64,65,66,67,68,69,70].

Therefore, appropriate management of the thalidomide dosage is necessary during its use. In previous studies, the therapeutic dose of thalidomide for IBD ranged from 50 to 400 mg/d. Considering the relationship between effect and adverse events and dosage, starting oral thalidomide doses of 50 mg/d are usually administered in adult patients and then subsequently increased according to the patient’s response and tolerance. Low-dose thalidomide (50–100 mg/d) was effective and tolerated for inducing and maintaining clinical remission in adult patients with active CD. The optimal clinical remission rate achieved was about 50% at weeks 12–24 [31]. Mucosal healing can be reasonably achievable with thalidomide. A retrospective study demonstrated that thalidomide is able to induce and maintain endoscopic remission in adult CD patients. The endoscopic remission rate in treated endoscopy active CD patients was 50%, and 71.4% achieved an endoscopy response [71]. In addition to the healing of luminal ulcers, thalidomide promoted the closure of perirectal or perianal fistulae, and a noticeable improvement occurred after 4 weeks of treatment with a dose of 200 mg/d [72].

##### Pediatric and Very-Early-Onset Inflammatory Bowel Disease

Thalidomide has also demonstrated promising therapeutic potential in pediatric and very-early-onset inflammatory bowel disease. Compared to adults, pediatric patients with IBD are more likely to have extensive intestinal involvement and rapid clinical progression [73,74]. They are also more likely to have a family history of IBD, suggesting a stronger genetic correlation for childhood-onset IBD [75,76]. An early age of onset indicates long-term impairment and a cumulative burden of conventional treatment. Moreover, resistance or intolerance to treatments is more common, and the recurrence rate is higher in children than in adults [77,78]. Multiple studies evaluated the efficacy of thalidomide in refractory pediatric IBD as a potential rescue therapy, although it is contraindicated in children because of unknown safety and effectiveness.

Lazzerini et al. conducted the first two RCTs in pediatric CD and UC patients. They randomized 28 children with CD to the thalidomide group (1.5 to 2.5 mg/kg/day) and 26 to the placebo group. In the thalidomide group, a higher proportion of children achieved clinical remission (46.4% vs. 11.5%), and more significant improvement was observed in this group after eight weeks [32]. In another pilot RCT focused on refractory UC, 26 children were randomized to the thalidomide (1.5 to 2.5 mg/kg/day) or placebo group. The clinical remission rate at week 8 was significantly higher with thalidomide treatment (83.3% vs. 18.8%) and was maintained for 135 weeks [33]. Considering the essential treatment goal of mucosal healing, the research team conducted a long-term analysis of the data from these two clinical trials. Treatment with thalidomide for 52 weeks led to clinical remission in 54.3% of patients, 75.3% showed mucosal healing, and 52.6% achieved histologic healing [79]. Moreover, pediatric patients with monogenic mutation (mainly the IL10RA mutation) tended to respond better to thalidomide [80].

Thalidomide has also demonstrated encouraging outcomes in several rare and challenging clinical cases. Only 5% of children with active UC encountered clinically have acute severe colitis [81]. Thalidomide was used as a rescue therapy in a pediatric acute severe UC case, who was nonresponsive to infliximab (IFX), allowing the patient to avoid a conventional (but invasive) colectomy [82]. Thalidomide was successfully used to treat a five-year-old de novo CD patient after ileal pouch–anal anastomosis for UC, which was the first report of using thalidomide to treat de novo CD [83]. CD-like disease may occur after allogeneic hematopoietic stem cell transplantation (allo-HSCT). A complete, stable clinical response was obtained with good tolerance eight weeks after the start of a thalidomide treatment in a case report. Mild peripheral neurotoxicity occurred five years later but disappeared entirely with a dose reduction [84].

The concept of very-early-onset inflammatory bowel disease (VEOIBD) was proposed in 2012 [85]. VEOIBD is a specific type of IBD, which mainly refers to IBD that starts before the age of 5 or 6 years and can begin as early as the neonatal period [86]. VEOIBD accounts for 6% to 15% of IBD cases in children and has the characteristics of an early onset, severe disease, difficult-to-control diarrhea, and a severe impact on growth and development, which are primarily associated with severe perianal lesions and mostly occur with occult genetic defects [87,88]. In one retrospective study, the remission rate after thalidomide use in VEOIBD reached 88.2%, and the mucosal healing rate reached 60% at 36 months. A significantly higher proportion of VEOIBD patients discontinued therapy due to lack of efficacy while adverse events were the main reason for discontinuation in pIBD patients. Peripheral neuropathy is the most common adverse event in VEOIBD (85.7%) [89]. No thalidomide treatment significantly lowered the probability of survival among infantile-onset IBD patients with an IL-10 signaling deficiency [90].

Most studies have found peripheral neuropathy to be the most common severe adverse event in pediatric patients. Thalidomide-induced peripheral neuropathy is generally mild and reversible, and is primarily due to axonal damage [91]. It is often associated with proximal weakness and may progress even after treatment discontinuation, which is referred to as the coasting phenomenon [92]. The cumulative dose seems to be the most relevant risk factor [80]. Lazzerini et al. reported that the minimum cumulative dose that caused neuropathy was 380 mg/kg in refractory pediatric CD patients and 332 mg/kg in UC patients [32,33]. Polymorphisms in the *ICAM1* and *SERPINB2* genes may be protective and involved in neuronal inflammation and repair [93]. According to the consensus guidelines of ECCO/ESPGHAN on the medical management of pediatric CD, thalidomide maintenance therapy can be an alternative for anti-TNF agent responders who do not tolerate or stop responding to biologic anti-TNF agents. A dose of 2 mg/kg was suggested for young children. Careful neurological and psychological examination and assessment of vibration sensitivity at regular intervals (6 months) is indicated [94].

##### Intestinal Behcet’s Disease

BD is a chronic recurrent systemic vasculitis typified by recurrent oral ulcers, genital ulcers, ocular inflammation, and skin damage, which can involve multiple organ systems throughout the body. Attempts to treat BD with thalidomide began in the 1980s, and the symptoms, including orogenital ulcerations, cutaneous lesions, and arthritis, improved after thalidomide treatment [95]. Hamuryudan et al. conducted an RCT to compare the effect of thalidomide (100 mg/d or 300 mg/d) and a placebo on mucocutaneous lesions in BD patients. The efficacy of 100 mg/d thalidomide was similar to that with a 300 mg/d dose [96]. In a multicenter retrospective cohort study, thalidomide was shown to be rapidly effective in severe recurrent aphthous stomatitis, and 85% (78/92) of the patients entered complete remission within a median of 14 days. For long-term maintenance, low-dose regimens appear to be effective and relatively well tolerated [97].

BD with gastrointestinal involvement is called intestinal BD, and its incidence is about 3–60%, with regional differences [98]. Intestinal Behcet’s disease is broadly categorized as an inflammatory bowel disorder. Intestinal BD can involve the entire gastrointestinal tract but most commonly affects the ileocecal region. Its clinical manifestations include abdominal pain and diarrhea, and colonoscopy usually reveals deep, large, well-defined ulcers. These ulcers can cause severe complications such as bleeding and perforation, resulting in high rates of disability and mortality. Intestinal BD mimics CD in clinical presentation and endoscopic manifestation, making a differential diagnosis challenging [99].

There are many reports of using thalidomide to treat gastrointestinal lesions in BD, with the initial dosage ranging from 100 to 400 mg/day [100,101,102]. A 24-year-old intestinal BD patient who underwent three operations due to recurrent perforation had her symptoms disappear within two weeks after starting thalidomide (100mg/day) and no other intestinal perforations occurred during the follow-up period of four months [34]. Seven juvenile-onset patients with severe recurrent intestinal involvement of BD showed dramatic improvements in their clinical symptoms with thalidomide therapy (2-3 mg/kg/day) and they successfully discontinued steroid therapy [35,36]. Even in infant patients with giant ulcers of the buccal mucosa, necrosis of the tongue, abdominal tenderness, and severe diarrhea due to BD, thalidomide promoted the recovery of the mucocutaneous lesions and gastrointestinal manifestations [103,104]. More recently, Hatemi et al. reported that one-fifth of their BD patients with gastrointestinal involvement were refractory to conventional treatment modalities but remission was achieved with TNF-alpha antagonists or thalidomide in about 75% of cases [105]. The 2018 update of the European League Against Rheumatism (EULAR) recommendations stated that for severe and/or refractory gastrointestinal involvement BD patients, monoclonal anti-TNF antibodies and/or thalidomide should be considered [15].

As mentioned above, pediatric patients with genetic abnormalities tended to respond better to thalidomide [80]. Besides IL-10-related monogenetic mutations in IBD, A20 haploinsufficiency can cause an early-onset autoinflammatory disease with BD-like clinical symptoms. Thalidomide effectively treated refractory gastrointestinal lesions caused by A20 haploinsufficiency in a 9-year-old boy and the only adverse effect was mild drowsiness [106,107]. According to a study on the largest cohort of patients with monogenic autoinflammatory diseases in China, thalidomide can also help reduce disease activity and inflammation, reduce the dosage of glucocorticoids needed, and improve clinical outcomes while being relatively safe [108]. The co-occurrence of myelodysplastic syndrome (MDS) with trisomy 8 and BD is another rare case, usually involving the intestinal tract. The shared pathogenic factor of BD and MDS is TNF-α. Therefore, TNF-α inhibitors are key therapeutic strategies, and, unsurprisingly, thalidomide markedly improved the symptoms in this patient population [109,110,111,112]. However, there is a lack of studies specifically addressing dose management of thalidomide in the treatment of intestinal Behcet’s disease.

#### 3.1.2. IgG4-Related Digestive Diseases

IgG4-related disease is an emerging systemic inflammatory disease that can affect virtually all organs, ultimately leading to fibrosis. IgG4-related disease can be categorized into four clinical phenotypes according to the organs involved. Among these, hepatic, pancreatic, and biliary manifestations represent about 31% of cases, retroperitoneal fibrosis and head and neck lesions each occur in roughly 24%, and Mikulicz’s syndrome without systemic manifestations accounts for around 22% [113]. Thus, the digestive system is the most frequently affected region in IgG4-related disease. A recent multicenter, randomized, double-blind, placebo-controlled trial demonstrated that thalidomide can effectively prevent relapse in IgG4-related disease, with the benefits outweighing its adverse effects [37]. The participants were randomized into two groups: glucocorticoids (GCs) plus thalidomide and GCs plus placebo. All patients initially received prednisone to induce remission, followed by gradual tapering until withdrawal. In addition, the patients were given thalidomide/placebo starting at 25 mg/day, which was then titrated up to a maximum of 75 mg/day depending on tolerability. The study indicated that clinical responses to thalidomide varied across the different phenotypes. In particular, thalidomide showed remarkable efficacy in the Pancreato-Hepato-Biliary phenotype. Within the placebo group, the relapse rate of this phenotype reached as high as 66.7%, whereas no relapses occurred in the thalidomide group. These findings suggest a potential preferential benefit of thalidomide for this subgroup. Among the participants receiving thalidomide, the most commonly observed adverse event was limb numbness, followed by dizziness, edema, and fatigue.

#### 3.1.3. Oral Mucosal Diseases

Given its anti-inflammatory properties, thalidomide has also been used off-label in the treatment of various oral mucosal diseases. Thalidomide has been shown to be effective in the treatment of recurrent oral ulcers in Behcet’s disease and is recommended by clinical guidelines [15,96]. In addition, its efficacy in treating recurrent aphthous stomatitis is also supported by clinical evidence, including improvements in complete and overall response rates, prolonged recurrence intervals, faster ulcer healing, and a reduction in both the number and size of lesions [14]. Zeng et al. concluded that thalidomide has a more sustained long-term effect than prednisone in prolonging the recurrence interval of recurrent aphthous stomatitis [114]. In terms of adverse effects, thalidomide treatment has been associated with an increased risk of peripheral neuropathy, thromboembolic events, constipation, and somnolence. However, most adverse effects can be minimized by a stepwise reduction in dosage. A recent dose optimization study concluded that a daily dose of 25 mg of thalidomide had a long-term effect on extending the recurrence interval of recurrent aphthous stomatitis with a good safety profile [38]. In addition, thalidomide has also applied in non-immune-related oral mucosal disorders; it has been shown to reduce the incidence and severity of radiation-induced oral mucositis in patients undergoing chemoradiotherapy for nasopharyngeal carcinoma, demonstrating a protective effect against mucosal injury [39]. There is evidence that thalidomide can suppress radiation-induced inflammation and apoptosis in oral epithelial cells by upregulating LZTS3 expression [115].

### 3.2. Non-Immune-Related Digestive Diseases

In addition to its immunomodulatory and anti-inflammatory properties, thalidomide also exhibits anti-angiogenic effects. This action underlies its therapeutic application in a range of non-immune-related digestive diseases, most notably in the management of gastrointestinal bleeding and various digestive malignant tumors.

#### 3.2.1. Gastrointestinal Bleeding

Thalidomide, due to its antiangiogenic properties, has emerged as a promising therapeutic option in managing various types of gastrointestinal bleeding. In recent years, both clinical studies and case reports have demonstrated its efficacy in various clinical conditions, including angiodysplasia-related bleeding and secondary GI bleeding due to underlying conditions such as radiation injury and portal hypertension.

##### Gastrointestinal Bleeding Due to Angiodysplasia

In 1994, thalidomide was found to have a potent antiangiogenic effect via downregulating VEGF [1]. VEGF expression is increased in patients with colonic angiogenesis, which plays a vital role in gastrointestinal angiodysplasia (GIAD) [116]. HIF-1α and HIF-2α induced angiogenesis in gastrointestinal vascular malformation and can be reversed by thalidomide, which exerts inhibitory effects upstream of VEGF [117]. Thalidomide has been used to successfully manage bleeding in patients with GIAD, mainly inherited bleeding disorders [118,119]. Ge et al. conducted an RCT and found that response rates in the thalidomide (25 mg 4 times/day for 4 m) and control groups (iron 100 mg/d for 4 m) were 71.4% and 3.7% (*p* < 0.001) in terms of refractory bleeding from gastrointestinal vascular malformations [40]. Furthermore, thalidomide was effective in reducing all the secondary endpoints, including rates of cessation of bleeding, blood transfusions, overall hospitalizations, and hospitalizations for bleeding. No severe adverse effects were observed. Minor side effects, including fatigue, constipation, and somnolence, were reported by 73% of the patients in the thalidomide group. In addition, serum levels of VEGF were consistently and significantly lower in the thalidomide group [40]. Small-intestinal angiodysplasia (SIA) is the most common cause of obscure gastrointestinal bleeding, which can also be resolved by thalidomide [120]. Chen et al. performed a randomized, double-blind, multicenter, placebo-controlled trial across China and supported the efficacy and safety of thalidomide in treating recurrent bleeding from SIA [26]. Besides targeting the VEGF-related pathway, thalidomide also targets EGFL6, inhibiting EGFL6/PAX6 axis-driven angiogenesis in small bowel vascular malformation disease [121]. Based on these results, it is reasonable to treat patients with recurrent or refractory bleeding with thalidomide when other treatments have failed. Several RCTs and meta-analyses have also drawn positive conclusions [122,123,124].

##### Secondary Gastrointestinal Bleeding

Gastrointestinal bleeding can also be secondary; by controlling this bleeding, thalidomide could be included in the treatment of the primary diseases. Radiation proctitis is one of the most frequently encountered late gastrointestinal sequelae of abdominopelvic radiation therapy. A patient with severe refractory hemorrhagic radiation proctitis requiring frequent blood transfusions was successfully treated with low-dose oral thalidomide [41]. According to rat model experiments, acute radiation-induced proctitis is related to micro-vessel endothelial cell injury, which could be attenuated with thalidomide [125]. Gastrointestinal bleeding is also a complication after kidney transplantation due to immunosuppressant use. Heo et al. presented a case of refractory small bowel bleeding treated successfully with thalidomide after multiple failed attempts using conventional treatment [42]. Thalidomide also showed beneficial effects and reversion of vascular lesions in a patient with bleeding portal hypertensive gastropathy and enteropathy that caused transfusion-dependent anemia [43,126]. Apart from the direct inhibition of angiogenesis, thalidomide can reduce portal hypertension by inhibiting TNF-α synthesis and reducing nitric oxide production, blunting the development of hyperdynamic circulation [127].

#### 3.2.2. Digestive Malignant Tumors

Thalidomide has been investigated for its therapeutic potential in digestive cancers, particularly hepatocellular carcinoma and colorectal cancer. Transcatheter arterial chemoembolization (TACE) and thalidomide have been used in the treatment of primary hepatocellular carcinoma. Compared to TACE alone, thalidomide combined with TACE has better clinical efficacy and tolerable adverse events [46]. In recent years, a combination regimen for hepatocellular carcinoma has been proposed, which consists of thalidomide, carmofur, and compound mylabris capsules, named the TCC cocktail. The three components of the TCC cocktail possess distinct antitumor mechanisms. A recent study demonstrated that the TCC cocktail may exert effective anti-hepatocellular carcinoma activity by inducing the SAMD4B-APOA2-PD-L1 axis to inhibit tumor immune evasion [128]. A randomized, open-label, multicenter clinical trial provided further evidence that the combination of TACE and the TCC cocktail is well tolerated and can significantly improve clinical outcomes in patients with unresectable hepatocellular carcinoma [47]. According to a single-center study, combining thalidomide with degraded first-line palliative oxaliplatin plus capecitabine chemotherapy showed notable efficacy in elderly patients with high-risk stage II/III colon or rectal cancer following surgery for metastatic colorectal cancer [45]. Another study reported that the addition of thalidomide to first-line oxaliplatin and capecitabine therapy significantly improved the disease control rate in patients with metastatic colorectal cancer, although it was associated with an increased incidence of constipation [44].

## 4. Strategies for the Clinical Use of Thalidomide

The current applications of thalidomide are largely confined to specific settings, often involving refractory cases. In most indications, thalidomide remains an off-label therapy, and its use is mainly supported by small-scale clinical studies or case series. Most of the clinical evidence comes from studies in inflammatory bowel disease, where thalidomide has been used as an alternative or salvage therapy, or in combination with other agents to enhance efficacy and reduce toxicity. This section summarizes the key clinical strategies, including these three approaches. Although the current data are primarily derived from IBD, these therapeutic strategies may hold potential for broader application in other disease contexts where inflammation, immune dysregulation, or abnormal angiogenesis play a key role.

### 4.1. Alternative Therapy

Biological therapy has dramatically improved the prognosis and regression of immune-related enteropathy. However, anti-TNF-α treatment in clinical application still faces many problems, such as failure to respond, intolerability, and enormous economic burdens. Thalidomide is an inexpensive agent and has anti-TNF-α effects. The incidence of adverse reactions in clinical use is high but these reactions are mild and generally tolerated by patients, and most patients can be safely treated under close supervision. Despite the accumulation of clinical experience and research evidence over more than 20 years, thalidomide is not used as a first-line therapy given the high risk of serious adverse effects and severe congenital disabilities. Most studies chose refractory patients as participants. According to guideline recommendations, thalidomide should mainly be used as an alternative or salvage treatment in exceptional circumstances when corticosteroid dependence or resistance occurs, and biological agents are ineffective or intolerable, or, rarely, in patients with delayed hypersensitivity responses to infliximab [72,129]. In addition to its anti-TNF-α effect, thalidomide has a variety of other mechanisms of action, including inhibition of angiogenesis, which may be one of the reasons why thalidomide remains effective in various refractory conditions. Angiogenesis can enhance the immune cell population in chronic inflammatory diseases, including IBD, by delivering oxygen and nutrients [130,131]. The pharmacological mechanism through which thalidomide inhibits angiogenesis also plays a role in CD. In pediatric CD patients, thalidomide can suppress the effect of angiogenic factors by targeting VEGF and Ang-2 [132].

### 4.2. Salvage Therapy

New infections or activation or exacerbation of latent infections are among the most critical issues to be aware of when using biological and potent immunosuppressive agents. Analyses of patients with gastrointestinal Behcet’s disease in Japan receiving TNFαi biologics in 2021 revealed that 40% experienced severe adverse events, including pneumonia (32%), gastrointestinal adverse events (15%), sepsis (5%), and intestinal perforation (2%) [133]. In an ulcerative intestinal tuberculosis case that occurred as a complication of treatment with infliximab for intestinal BD, oral thalidomide combined with anti-tuberculous drugs stabilized the patient’s condition [134]. A similar therapeutic effect was also observed in pediatric and young adult CD patients with evidence of tuberculosis [135,136]. Even in an CD patient with hematogenous disseminated tuberculosis secondary to infliximab injection, the clinical condition significantly improved with thalidomide and glucocorticoid treatment [137]. These cases suggest that thalidomide can be used to avoid exacerbating infections. Biological therapies have been associated with an increased incidence of infections, especially tuberculosis. Thalidomide is an oral immunomodulatory agent with anti-TNF-α properties. It stimulates T lymphocytes, with a more significant effect on CD8^+^ than on CD4^+^ T cells, contributing to the protective immune response to tuberculosis infection [138,139].

### 4.3. Combination Therapy

Some studies have explored combination or sequential therapy options. The efficacy of monotherapy can be enhanced by combining thalidomide with other conventional treatments while reducing the dose of thalidomide. Li et al. reviewed 122 consecutive CD patients who stopped responding to azathioprine (AZA) therapy and switched to a combination therapy of thalidomide and AZA. The proportions of patients in remission at 12, 24, and 36 months were 85.1%, 78.3%, and 70.1%, respectively. The endoscopic remission rate reached 63.6%, and 23.6% achieved mucosal healing. Adverse events occurred in 62 (50.8%) patients. Acroanesthesia was the most common side effect (17.2%), followed by constipation (10.7%). Seven patients had to stop taking thalidomide but the side effects were relieved after discontinuation [140]. Low-dose thalidomide combined with mesalazine can have significant effects (11/14) in treating refractory UC with limited adverse events, suggesting potential synergistic effects [141]. In chronically active and fistulizing refractory CD, thalidomide appears to be an effective and relatively safe drug that can be used as maintenance therapy after remission has been achieved with infliximab [142]. Thalidomide has been suggested as a concomitant therapy with IFX for intestinal BS patients [143]. Bao et al. found that when thalidomide was administrated to intestinal BS patients treated with IFX, the rates of clinical remission decreased (OR: 0.13; 95% CI: 0.02–0.78; *p* = 0.025) [144]. Apart from these traditional medications, treatment strategies targeted at gut microbiota have also attracted attention from IBD researchers. Combining multi-donor fecal microbiota transplantation (FMT) with thalidomide optimized the clinical response and mucosal healing rate in hormone-dependent UC patients [145]. The TCC cocktail for hepatocellular carcinoma mentioned above represents another example of a combination therapy that includes thalidomide. The advantage of this regimen lies in the distinct mechanisms of action of its components, which may exert synergistic antitumor effects. In addition, the adverse effects of the individual agents may offset one another, thereby potentially improving the overall safety profile.

Thus, the therapeutic status of thalidomide in digestive disorders is not clear. More well-designed clinical studies are needed to explore the strategies where thalidomide is used as a mono-, alternative, salvage, enhancement, or adjunctive therapy in combination with traditional medications.

## 5. Adverse Effects of Thalidomide and Strategies to Mitigate Its Toxicity

### 5.1. Adverse Effects

The most severe adverse event associated with thalidomide is teratogenicity. In addition to teratogenicity, which is mostly preventable, the adverse effects of thalidomide include peripheral neuropathy, drowsiness, constipation, thrombosis, neutropenia, severe skin reactions, hypotension, and bradycardia [146]. Among these, peripheral neuropathy is a common reason for treatment discontinuation. The risk of neuropathy is closely related to the daily dose [147]. Thromboembolic events are more likely in patients with risk factors such as older age, obesity, prior history of thrombosis, or corticosteroid use [148,149]. Constipation and somnolence are generally mild and dose-related, reflecting thalidomide’s effects on the nervous system [149]. These adverse reactions are associated with dose accumulation and may resolve after discontinuation of the drug. The mechanisms through which thalidomide causes its major adverse effects are summarized in Table 2. Thalidomide-induced teratogenicity is primarily attributed to its anti-angiogenic properties, as proper angiogenesis is essential for organ and limb development during embryogenesis [150]. Similarly, its anti-angiogenic effects may contribute to peripheral neuropathy through secondary ischemia and hypoxia of nerve fibers. Moreover, thalidomide may reduce neuronal cell survival due to its downregulation of TNF-α and inhibition of NF-κB, resulting in the dysregulation of neurotrophins and their receptors and the subsequent acceleration of neuronal cell death [151]. Thalidomide may exert a prothrombotic effect by reducing anticoagulant protein levels and enhancing platelet aggregation and procoagulant activity; however, the exact underlying mechanism remains unclear [152].

### 5.2. Strategies for Optimizing Thalidomide Therapy and Reducing Its Toxicity

The adverse effects associated with thalidomide are the most critical factor limiting its clinical use. Lowering the drug dose, combining it with other medications, and monitoring for possible adverse reactions are commonly used to manage the side effects in clinical practice. Due to the CRBN-centered, single-target but pleiotropic model of thalidomide action, rational structural modifications and advanced drug delivery strategies could also help mitigate adverse effects by reducing thalidomide’s off-target activity and enhancing its therapeutic precision.

#### 5.2.1. Dose and Risk Management of Thalidomide in Clinical Practice

Owing to its teratogenicity, thalidomide use was first restricted by the S.T.E.P.S. program and later regulated by a program called the Thalidomide REMS (Risk Evaluation and Mitigation Strategy) [11,153]. In clinical use, physicians focus on controlling the disease and reducing adverse reactions by lowering the drug dose, combining it with other medications, and monitoring for possible adverse reactions. Common adverse reactions such as somnolence can be mitigated by advising patients to take the medication at bedtime, and peripheral neuropathy symptoms like numbness in the hands and feet can be partially alleviated with symptomatic supplementation such as mecobalamin. Furthermore, a machine learning algorithm has been developed to predict thalidomide-induced peripheral neuropathy based on 18 clinical features and 14 genetic variables, providing new ideas for identifying the patients that are likely to benefit from this drug therapy [154].

#### 5.2.2. Structural Modifications to Thalidomide to Reduce Toxicity

**Figure 3:**
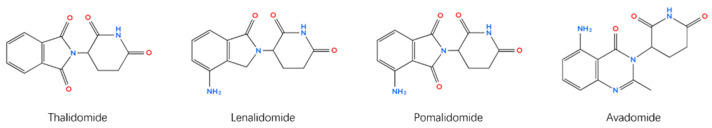
Chemical structures of thalidomide and its analogs.

New immunomodulatory anticancer thalidomide analogs have been designed and synthesized, primarily to enhance the treatment of multiple myeloma (Figure 3) [155]. These analogs have the common structural feature of a glutarimide moiety attached to a heteroaromatic nucleus. Replacing the phthalimide moiety with other aromatic nuclei improved the activity and toxicity profiles. Lenalidomide, pomalidomide, and avadomide are concrete examples of the potential of this approach [156]. Compared to thalidomide, lenalidomide is more effective and safer, with less neurotoxicity but more severe hematologic adverse effects [157]. Pomalidomide is more potent and less toxic than thalidomide and lenalidomide [158]. Hybridizing the immunomodulatory glutarimide with known anticancer nuclei using different linkers is the latest attempt to achieve the ideal immunomodulatory anticancer effect [159,160]. However, these new analogs have higher costs, which is the main reason why they cannot fully replace thalidomide [157].

#### 5.2.3. Targeted and Novel Delivery Systems

Promoting mucosal healing of intestinal lesions is the critical goal of treatment. Therefore, reducing the drug concentration in the blood circulation and achieving targeted and sustained drug action in the gastrointestinal tract is another way to optimize thalidomide’s clinical use. Many researchers have attempted to increase the therapeutic effects of thalidomide and reduce systematic exposure using a specially designed drug delivery system. Most of the thalidomide drug delivery studies have focused on intravenous administration. Poly (ethylene glycol) methyl ether-functionalized graphene oxide bearing two commonly used drugs (lidocaine and thalidomide) exhibited synergistic effects on neuropathic pain and inhibition of proinflammatory cytokines in vitro and in vivo [161]. Another study incubated exosomes mixed with liposomes to form hybrid exosomes to deliver thalidomide and found that this approach significantly increased its solubility and inhibited TNF-induced expansion and proliferation of T regulatory cells [162]. In addition, thalidomide remodels abnormal tumor vasculature into a more normal architecture, improving the delivery of conventional chemotherapeutic agents in solid tumors [163]. To increase the efficacy of oral thalidomide in treating digestive disorders, Fakhoury et al. used alginate-poly-L-lysine-alginate microcapsules to contain thalidomide and successfully reduced intestinal inflammation [164]. Meng et al. designed polydopamine-coated thalidomide nanocrystals, which could reduce disease activity by inducing polarization of macrophages towards the M2 phenotype, reducing proinflammatory cytokines levels, and normalizing blood vessels in colitis induced by dextran sulfate sodium salt (DSS). In the test of toxicity, no histological changes were observed in the heart, liver, spleen, lung, or kidney, and no significant myelosuppression occurred [165]. Another similar gastric acid-resistance and pH-switchable controlled release system enhanced the oral bioavailability of thalidomide and has the potential to attenuate radiation-induced gastrointestinal syndrome [166].

## 6. Strengths and Limitations

**Figure 4:**
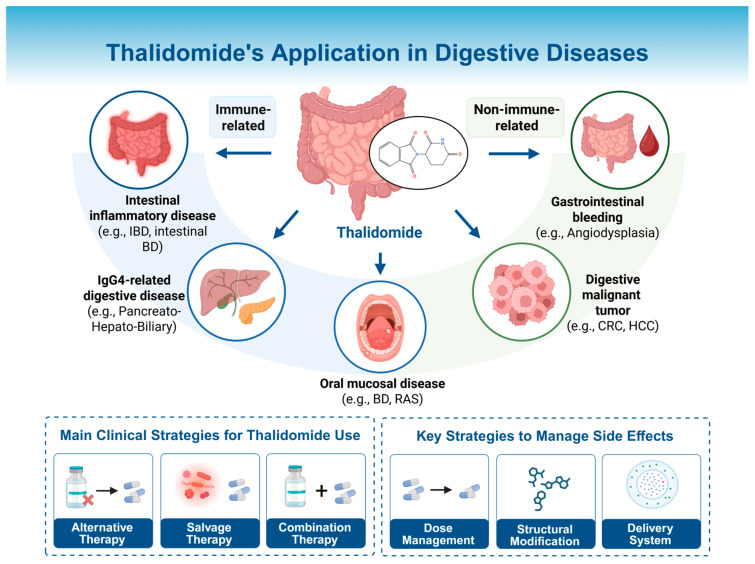
Thalidomide’s application in digestive diseases. Thalidomide has been applied across a range of digestive disorders, including intestinal inflammatory diseases, IgG4-related digestive diseases, oral mucosal diseases, gastrointestinal bleeding diseases, and digestive malignancies. The clinical use strategies are categorized into three main approaches: alternative, salvage, and combination therapies. The optimization strategies to reduce adverse effects include dose and risk management in clinical practice, structural modification of the molecule, and the development of advanced drug delivery systems. IBD: inflammatory bowel disease; BD: Behcet’s disease; RAS: recurrent aphthous stomatitis; CRC: colorectal cancer; HCC: hepatocellular carcinoma. Created in BioRender. Chen, W. (2025) https://BioRender.com/tpm9ajm.

Consistent with previous reviews [167,168], this review acknowledges the promising potential and expanding application of thalidomide. First, it placed a greater emphasis on clinical practicality, focusing on real-world therapeutic strategies such as alternative, salvage, and combination therapies, along with practical dosing guidance. Second, it not only summarized the recent advances in thalidomide’s clinical applications in gastrointestinal diseases but also, for the first time, incorporated evidence on its use in digestive gland-related conditions from the latest studies. Third, it highlighted strategies for mitigating thalidomide-associated adverse effects, including dosage selection and adjustment, structural modification, and optimized drug delivery systems (Figure 4). Nonetheless, the evidence on the efficacy of thalidomide’s therapeutic strategies remains limited and is largely derived from intestinal inflammatory diseases. Further high-quality studies are warranted to validate these findings and optimize clinical protocols.

## 7. Conclusions

Thalidomide is a typical example of the “new use of old drugs,” from the original notorious molecule to an essential immunomodulating, anti-inflammatory, and antitumor agent. This review focused on the use of thalidomide to treat digestive disorders and demonstrated its large range of potential application scenarios. In particular, in immune-related intestinal lesions, whose incidence has shown a significant increase, thalidomide has become an important therapeutic option for refractory patients. Considering that thalidomide has anti-TNFα effects like those of biologics but with a more convenient and economically less costly oral route of administration, future studies should focus on determining thalidomide’s priority and dosing strategy, and identifying the appropriate target population for the treatment of immune enteropathies.

From a regulatory perspective, specific risk management programs such as the REMS in the United States and similar frameworks in Europe and Asia should include strict pregnancy prevention and patient education [169]. Currently, the officially approved indications are limited to erythema nodosum leprosum and multiple myeloma, as mentioned above, whereas its application in inflammatory bowel disease and other gastrointestinal conditions remains off-label, mainly guided by evidence from small-scale clinical trials and recent RCTs. In the existing clinical guidelines and consensus statements, the recommendations for thalidomide in digestive diseases remain limited or absent in most application scenarios (Table 1). Nevertheless, several randomized controlled trials exploring its use in different gastrointestinal conditions have been published in recent years, providing emerging supportive evidence. Given thalidomide’s teratogenicity, its repositioning for other indications involves ethical complexities. The use of thalidomide requires strict adherence to risk management programs such as the REMS, along with comprehensive informed consent. Clinicians must carefully balance the potential therapeutic benefits against these serious risks, particularly in severe or refractory cases where alternative treatments are limited. Furthermore, ethical oversight is essential to ensure that off-label or investigational use of thalidomide is conducted responsibly and in accordance with regulatory standards.

**Figure 5:**
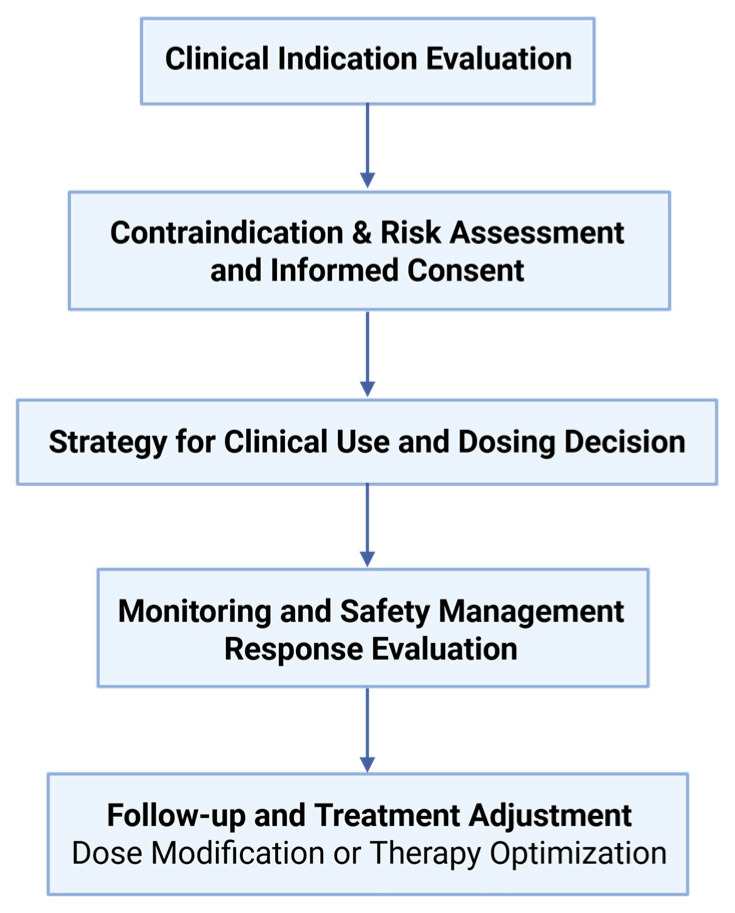
Clinical process of thalidomide administration. This flowchart outlines the stepwise approach to ensure rational and safe thalidomide use, from indication and risk assessment to therapeutic monitoring and evaluation.

As repeatedly mentioned in several previous studies and in this review, the most critical factor limiting the long-term use of thalidomide in patients is the high incidence of adverse reactions. In terms of clinical application, the occurrence of adverse reactions should be comprehensively and systematically monitored by patients, physicians, hospitals, and regulatory organizations. Necessary symptomatic treatment should be given, and the dosage and usage of thalidomide should be appropriately adjusted and, if necessary, discontinued. The clinical decision-making framework for thalidomide use is illustrated in Figure 5, outlining key steps from indication assessment to response evaluation. In terms of research, the pharmacokinetics and pharmacodynamics of thalidomide, especially the mechanisms underlying its adverse effects, still need to be explored in depth. Based on the new proposed indications, a comprehensive update of this old drug with a new structure, new dosage form, and new mode of administration should be carried out.

## Figures and Tables

**Table 1:** Thalidomide in digestive diseases.

Disease		Common Adverse Effects	Grade of Recommendations in Guidelines or Consensus ^a^	Dose Used in Clinical Studies	Dose Used in Case Reports
Inflammatory intestinal diseases	IBD in adults	Drowsiness, constipation, rash, and peripheral neuropathy	——	50–100 mg/d [31]	——
	Pediatric and very-early-onset IBD	Peripheral neuropathy	——	1.5–2.5 mg/kg/d * [32,33]	——
	Intestinal Behcet’s disease	Peripheral neuropathy	C [15]		100 mg/d [34]; 2–3 mg/kg/d (in children) [35,36]
IgG4-related disease	IgG4-related digestive disease	Limb numbness, dizziness, edema, and fatigue	——	25–75 mg/d * [37]	
Oral mucosal diseases	Recurrent aphthous stomatitis	Peripheral neuropathy, thromboembolic events, constipation, and somnolence	——	25 mg/d * [38]	——
	Radiation-induced oral mucositis	Dizziness and constipation	——	75 mg/d * [39]	——
Gastrointestinal bleeding	Gastrointestinal bleeding due to angiodysplasia	Constipation, somnolence, limb numbness, peripheral edema, dizziness, and elevated liver-enzyme levels	——	50 mg/d *; 100 mg/d * [26,40]	——
	Hemorrhagic radiation proctitis	——	——	——	50–100 mg/d [41]
	GI bleeding after kidney transplantation	——	——	——	100 mg/d [42]
	Bleeding portal hypertensive gastropathy and enteropathy	——	——	——	100 mg/d [43]
Digestive malignant tumors	Colorectal cancer	Constipation and lethargy	——	200 mg/d * [44]	100 mg/d [45]
	Hepatocellular carcinoma	Rash	——	200–400 mg/d * [46]; 50 mg/d (TCC cocktail) * [47]	——

^a^ Grade of recommendation is based on evidence: A, category I evidence; B, category II evidence or extrapolated recommendations from category I evidence; C, category III evidence or extrapolated recommendation from category I or II evidence; D, category IV evidence or extrapolated recommendation from category II or III evidence. Level of evidence indicates evidence from meta-analysis of RCTs (IA); at least one RCT (IB); at least one controlled study without randomization (IIA); at least one type of quasi-experimental study (IIB); descriptive studies, such as comparative studies, correlation studies, or case–control studies (III); and expert committee reports or opinions and/or clinical experience of respected authorities (IV). * Evidence from randomized controlled trials; IBD: inflammatory bowel disease; GI: gastrointestinal bleeding; TCC cocktail: thalidomide, carmofur, and compound mylabris capsules.

**Table 2:** Proposed mechanisms through which thalidomide causes its adverse effects.

Adverse Effect	Proposed Mechanism
Teratogenicity	Anti-angiogenic activity interferes with embryonic vascular development, leading to ischemia of developing tissues and limb buds
Peripheral neuropathy	Secondary ischemia and hypoxia of nerve fibers due to anti-angiogenic effects; downregulation of TNF-α and inhibition of NF-κB reduce neuronal survival and neurotrophic signaling
Thromboembolic events	Enhanced platelet aggregation and procoagulant activity
Constipation	Autonomic neuropathy affects intestinal motility

## Data Availability

No new data were created or analysed in this study. Data sharing is not applicable to this article.

## Abbreviations

The following abbreviations are used in this manuscript:

VEGF	vascular endothelial growth factor
GI	gastrointestinal
IBD	inflammatory bowel disease
BD	Behcet’s disease
IgG4	Immunoglobulin G4
TNF	tumor necrosis factor
IFN	interferon
IL	interleukin
CRBN	cereblon
UPS	ubiquitin–proteasome system
UC	ulcerative colitis
CD	Crohn’s disease
RCT	randomized controlled trial
allo-HSCT	allogeneic hematopoietic stem cell transplantation
VEOIBD	very-early-onset inflammatory bowel disease
EULAR	European League Against Rheumatism
MDS	myelodysplastic syndrome
GCs	glucocorticoids
GIAD	gastrointestinal angiodysplasia
SIA	small-intestinal angiodysplasia
TACE	transcatheter arterial chemoembolization
AZA	azathioprine
IFX	infliximab
FMT	fecal microbiota transplantation
DSS	dextran sulfate sodium salt
REMS	Risk Evaluation and Mitigation Strategy
